# The Role of Interleukin-15 Polymorphisms in Adult Acute Lymphoblastic Leukemia

**DOI:** 10.1371/journal.pone.0013626

**Published:** 2010-10-25

**Authors:** Dandan Lin, Chunliang Liu, Mengxing Xue, Rengyun Liu, Lan Jiang, Xiao Yu, Guangming Bao, Fang Deng, Mingjie Yu, Jiafu Ao, Yifeng Zhou, Depei Wu, Haiyan Liu

**Affiliations:** 1 Laboratory of Cellular and Molecular Tumor Immunology, Cyrus Tang Hematology Center, Department of Hematology, Jiangsu Institute of Hematology, The First Affiliated Hospital, Soochow University, Suzhou, China; 2 Thrombosis and Hemostasis Key Lab of the Ministry of Health, Soochow University, Suzhou, China; 3 Deparment of Biochemistry and Molecular Biology, Medical College of Soochow University, Suzhou, China; 4 Laboratory of Cancer Molecular Genetics, Medical College of Soochow University, Suzhou, Jiangsu, China; 5 The People's Hospital of Bozhou, Bozhou, China; University of Barcelona, Spain

## Abstract

**Background:**

Interleukin-15 (IL-15) plays important roles in the immune system and in the development of hematopoietic cells. Previous studies revealed that five SNPs in *IL-15*, rs10519612, rs10519613, rs35964658, rs17007695 and rs17015014, were significantly associated with childhood Acute Lymphoblastic Leukemia (ALL) treatment response. In adult ALL, the expression of IL-15 was also correlated with the immunophenotypes of ALL. Therefore, we hypothesize that SNPs of *IL-15* might also be associated with adult ALL.

**Methods and Findings:**

We genotyped the above five SNPs of *IL-15* gene by PCR-RFLP assays in adult ALL case-control studies. The current study included 121 adult ALL patients and 263 healthy controls. *IL-15* genotypes and haplotypes were determined and the associations with the risk of ALL were analyzed by logistic regression. SNPs rs10519612 and rs17007695 were significantly associated with ALL (*P* = 0.013 and *P* = 0.001). We observed a 2-fold and 2.4-fold excess risk of developing ALL for the rs10519612 CC and rs17007695 TC genotype carriers compared with non-carriers, respectively. Haplotype analysis revealed that haplotypes ACAC, CAGT and CCAT were significantly associated with adult B-ALL, while haplotype CCAT conferred susceptibility to T-ALL.

**Conclusion:**

These findings suggest that IL-15 gene polymorphisms are significantly associated with ALL in adult Chinese population.

## Introduction

The characteristic of acute lymphoblastic leukemia (ALL) is the clonal proliferation and accumulation of immature lymphoid cells [1]. Although 80% of children with ALL are cured with contemporary treatments, many patients still develop serious acute and late complications owing to the side effects of the treatments [2], [3]. Furthermore, the survival rate for adults with ALL remains below 40% [4]–[9]. The relatively poor outcome in adult ALL has been explained by an increased frequency of high-risk molecular subtypes with more aggressive clinical behavior and greater drug resistance, poorer tolerance and compliance with treatment, and less effective treatment regimens compared with childhood ALL [6], [8], [9]. Now many of techniques are used in diagnosing the biological and clinical differences of leukemic cells, including the acquisition of new chromosomal abnormalities, gene mutations, and reduced responsiveness to chemotherapeutic agents [10]–[13]. DNA copy number abnormalities (CNAs) and loss of heterozygosity (LOH) in cancer can be genome-wide analyzed by Single Nucleotide Polymorphism (SNP) arrays, which has provided important insights into the pathogenesis of newly diagnosed ALL [14].

Interleukin-15 (IL-15) was found to share many biological activities with IL-2, which is a pleiotropic cytokine of the 4-α-helix bundle cytokine family [15], [16]. IL-15 has many functions in immune system, including the activation of the proliferation and differentiation of natural killer (NK), T, and B cells, as well as the maintenance of memory T cells [15]–[20]. IL-15 appears to play a major role in the hematopoietic development controlling the survival, proliferation, and differentiation of both normal and leukemic progenitors, and can promote pathogenesis of leukemic cells [21]–[25]. Some studies have demonstrated that IL-15 can promote tumor cell growth, expansion, and organ infiltration of malignancies [23]–[27]. With respect to the potential clinical and therapeutic relevance of IL-15 in hematopoietic malignancies, high levels of IL-15 affect the initial response to therapy and have a relationship with malignant transformation, including organ infiltration and disease progression [26]–[30]. Five SNPs in *IL-15* gene identified by genome-wide scan significantly associated with childhood ALL treatment response [31]. So, IL-15 may play an important role in the development and progression of ALL.

Because of the possible roles of IL-15 in ALL development, we performed case–control studies to investigate the association between the five SNPs in human *IL-15* gene and adult ALL susceptibility in a Chinese population.

## Materials and Methods

### Study Population

The study group consisted of 121 Han Chinese patients with ALL, whose age ranged from 20 to 75. The group included 41 T-ALL patients and 80 B-ALL patients. These patients were recruited from March 2003 to May 2008 at the First Affiliated Hospital of Soochow University (Suzhou, China). Diagnosing ALL began with a medical history and physical examination, complete blood count and blood smears. Pathological examination, cytogenetics and immunophenotyping established whether the blast cells began from the B or T lymphocytes. All eligible patients diagnosed at the hospital during the study period were recruited, with a response rate of 94%. Population controls were cancer-free people living in the same region; they were selected from a nutritional survey conducted in the same period as the cases were collected. All control subjects had no history of cancer, and their age and gender frequency were matched to cases. The clinical characteristics of patients with acute lymphoblastic leukemia are shown in Table 1 [32].

**Table 1:** Clinical characteristics of patients with acute lymphoblastic leukemia.

		No.(121)	%
Gender			
	Male	66	54.5
	Female	55	45.5
Age at diagnosis, y			
	20–40	69	57
	41–60	44	36.4
	≥61	8	6.6
White blood cell count at diagnosis, /µl			
	<50000	58	47.9
	≥50000	63	52.1
Lineage			
	T-ALL	41	33.9
	B-ALL	80	66.1
Karyotype			
	Aberrant	67	55.4
	Normal	54	44.6
Molecular subtype			
	BCR-ABL	22	18.2
	E2A-PBX1	2	1.7
	MLL rearrangement	10	8.3
	No common translocations	87	71.9
Prognosis Risk Rank*			
	Low risk	8	6.6
	Intermediate risk	57	47.1
	High risk	56	46.3

*According to the risk stratification of adult ALL proposed by Hoelzer *et al* [32].

### Ethics Statement

The bone marrow samples of patients and blood samples of healthy individuals used in this study were part of the samples taken for clinical diagnostic tests. Since no extra amount of samples were collected from the study subjects, verbal consent was received from all patients and healthy control individuals. The study procedure was approved by the ethics committee of Soochow University.

### DNA extraction and genotyping analysis

Genomic DNA was extracted from 2 ml frozen whole blood or bone marrow using the RelaxGene Blood DNA system according to the manufacturer's protocol (Tiangen, China). A polymerase chain reaction-restriction fragment length polymorphism (PCR-RFLP) assay was used to detect polymorphisms. The sequences of the primers (Shenggong, China) for the PCR and annealing temperatures for the PCRs are listed in Table 2.

**Table 2:** Primers and Restriction Endonucleases used for genotyping *IL-15* gene.

SNP ID	Primer sequences	Product	Annealing	Restriction
		Length (bp)	temperature(°C)	Endonucleases
rs10519612	F: 5′-CTCAATGTCCTTAACCCATTATTCGA-3′	137	56.1	BspT104 I
	R: 5′-CGTTTGAACCATATGGTGAGGTCT-3′			
rs10519613	F: 5′-TAGACATAACAAAACACTCGGCATTT-3′	122	59.3	Dra I
	R: 5′-CTCAATGACATTTTTCTGCCTTCA-3′			
rs35964658	F: 5′-GTTCTGAAACTTTTCATCAAATGAGC-3′	149	62.4	Hha I
	R: 5′-CTAGCCTTACTGGGGAAAATGG-3′			
rs17007695	F: 5′-GTCTTTCTCATGGTCCTCATTGA-3′	126	56.1	BspT104 I
	R: 5′-AAAATTTACAAATGTGTGATTTTCGA-3′			
rs17015014	F: 5′-CGGACTGCTGGGTCTAAGAAGCTA-3′	119	62.4	Xsp I
	R: 5′-GTCTGACTCATCAGCCAACACCC-3′			

F = Forward Primer; R = Reverse Primer.

The polymorphic region was amplified by PCR in a 25 µl reaction solution containing 50 to 100 ng genomic DNA, 1×Taq PCR buffer, 1.5 mM MgCl_2_, 0.2 mM dNTPs, 5 U Taq DNA polymerase (Fermentas, EU) and 0.2 uM of each primer. PCR products were digested 3 hrs with restriction enzymes (Takara, Japan) according to the manufacturer's protocol and analyzed by 3% agarose gel electrophoresis. The length of PCR products and restriction enzymes are shown in Table 2.

### Statistical analysis

SNP allele frequencies were tested against departure from Hardy-Weinberg equilibrium (HWE) before analysis. χ^2^ tests and independent-samples t test were used to compare the difference in gender and age between cases and controls. Comparisons of the distributions of the allele, genotype and haplotype frequencies were performed using the χ^2^ test. The relative risk associated with rare alleles was estimated as an odds ratio (OR) with a 95% confidence interval (CI). The *P* values of OR and 95% CI indicated in the text are used to estimate the significance of the contribution of corresponding genotype to disease risk. The *P* values of genotypes indicated in the tables are used to estimate the significance of the distribution of genotype between cases and controls. A *P* value less than 0.05 was considered statistically significant. The statistical software package SPSS v. 17.0 was used for analyses. Haplotype frequencies for pairs of alleles as well as Linkage Disequilibrium (LD) coefficients *D*' = *D*/*D*_max_ and *r* ^2^ values for pairs of the most common alleles at each locus were estimated using the SHEsis program (http://analysis.bio-x.cn/myAnalysis.php)[33], [34].

## Results

### rs10519612 and rs17007695 of *IL-15* gene were significantly associated with adult ALL

There was no statistical difference in the age and gender distribution between ALL patients and controls (*P* = 0.381, *P* = 0.111). The allele and genotype distributions for five SNPs of *IL-15* among cases and controls were summarized in Table 3. The genotype frequencies of these polymorphisms were in Hardy-Weinberg equilibrium (*P*>0.05) in controls in this study. When the genotype frequencies were compared between cases and controls, rs10519612 and rs17007695 of *IL-15* gene showed statistically significant association with the disease. The distribution of rs10519612 and rs17007695 genotypes were significantly different between cases and controls (*P* = 0.013; *P* = 0.001) and the rs10519612 CC genotype and the rs17007695 TC genotype were more prevalent among the patients. Increased risks for ALL were found for the homozygous variant CC (OR 1.96, 95%CI 1.06–3.61) and TC (OR 2.38, 95%CI 1.43–3.97). The rs10519612 C allele and rs17007695 C allele carrier frequencies were higher in patients than in controls (*P* = 0.042, OR 1.37, 95%CI 1.01–1.87; *P* = 0.041, OR 1.38, 95%CI 1.01–1.88).

**Table 3:** Genotype and allele frequencies of *IL-15* SNPs among ALL cases and controls and associations with risk of ALL.

SNP		ALL Subjects (Cases/controls)			T-ALL Subjects (Cases/controls)			B-ALL Subjects (Cases/controls)		
		n (%)	OR (95% CI)*	*P*	n (%)	OR (95% CI)*	*P*	n (%)	OR (95% CI)*	*P*
rs10519612										
Genotype	AA	38/91 (31.4/34.6)	1.00		10/91 (24.4/34.6)	1.00		28/91 (35.0/34.6)	1.00	
	AC	51/135 (42.1/51.3)	0.88 (0.53–1.45)		19/135 (46.3/51.3)	1.25 (0.55–2.84)		32/135 (40.0/51.3)	0.74 (0.42–1.32)	
	CC	32/37 (26.4/14.1)	1.96 (1.06–3.61)&	0.013	12/37 (29.3/14.1)	2.68 (1.05–6.83)&	0.041	20/37 (25.0/14.1)	1.62 (0.81–3.26)	0.049
Allele	A	127/317 (52.5/60.3)	1.00		39/317 (47.6/60.3)	1.00		88/317 (55.0/60.3)	1.00	
	C	115/209 (47.5/39.7)	1.37 (1.01–1.87)	0.042	43/209 (52.4/39.7)	1.67 (1.05–2.67)	0.030	72/209 (45.0/39.7)	1.24 (0.87–1.77)	0.236
rs10519613										
Genotype	CC	40/103 (33.1/39.2)	1.00		12/103 (29.3/39.2)	1.00		28/103 (35.0/39.2)	1.00	
	CA	62/131 (51.2/49.8)	1.17 (0.73–1.89)		23/131 (56.1/49.8)	1.46 (0.69–3.10)		39/131 (48.7/49.8)	1.05 (0.60–1.83)	
	AA	19/29 (15.7/11.0)	1.56 (0.78–3.11)	0.315	6/29 (14.6/11.0)	1.48 (0.50–4.40)	0.450	13/29 (16.3/11.0)	1.49 (0.68–3.28)	0.436
Allele	C	142/337 (58.7/64.1)	1.00		47/337 (57.3/64.1)	1.00		95/337 (59.4/64.1)	1.00	
	A	100/189 (41.3/35.9)	1.26 (0.92–1.72)	0.152	35/189 (42.7/35.9)	1.33 (0.83–2.13)	0.238	65/189 (40.6/35.9)	1.22 (0.85–1.75)	0.282
rs35964658										
Genotype	AA	31/85 (26.1/32.3)	1.00		10/85 (24.4/32.3)	1.00		21/85 (26.9/32.3)	1.00	
	AG	67/142 (56.3/54.0)	1.25 (0.76–2.08)		26/142 (63.4/54.0)	1.48 (0.68–3.25)		41/142 (52.6/54.0)	1.12 (0.62–2.02)	
	GG	21/36 (17.6/13.7)	1.52 (0.77–3.00)	0.368	5/36 (12.2/13.7)	1.09 (0.34–3.44)	0.511	16/36 (20.5/13.7)	1.69 (0.79–3.64)	0.298
Allele	A	129/312 (54.2/59.3)	1.00		46/312 (56.1/59.3)	1.00		83/312 (53.2/59.3)	1.00	
	G	109/214 (45.8/40.7)	1.23 (0.91–1.68)	0.185	36/214 (43.9/40.7)	1.14 (0.71–1.83)	0.582	73/214 (46.8/40.7)	1.28 (0.90–1.84)	0.175
rs17007695										
Genotype	TT	27/104 (22.3/39.5)	1.00		9/104 (22.0/39.5)	1.00		18/104 (22.5/39.5)	1.00	
	TC	80/124 (66.1/47.1)	2.38 (1.43–3.97)&		25/124 (61.0/47.1)	2.28 (1.01–5.17)&		55/124 (68.8/47.1)	2.40 (1.32–4.36)&	
	CC	14/35 (11.6/13.3)	1.40 (0.65–3.01)	0.001	7/35 (17.1/13.3)	2.19 (0.74–6.47)	0.095	7/35 (8.7/13.3)	1.02 (0.39–2.69)	0.003
Allele	T	134/332 (55.4/63.1)	1.00		43/332 (52.4/63.1)	1.00		91/332 (56.9/63.1)	1.00	
	C	108/194 (44.6/36.9)	1.38 (1.01–1.88)	0.041	39/194 (47.6/36.9)	1.55 (0.97–2.48)	0.064	69/194 (43.1/36.9)	1.30 (0.91–1.86)	0.155
rs17015014										
Genotype	GG	30/75 (24.8/28.5)	1.00		9/75 (22.0/28.5)	1.00		21/75 (26.3/28.5)	1.00	
	GC	67/133 (55.4/50.6)	1.22 (0.73–2.05)		24/133 (58.5/50.6)	1.43 (0.63–3.27)		43/133 (53.7/50.6)	1.12 (0.62–2.03)	
	CC	24/55 (19.8/20.9)	1.07 (0.56–2.03)	0.659	8/55 (19.5/20.9)	1.20 (0.43–3.36)	0.600	16/55 (20.0/20.9)	1.03 (0.49–2.17)	0.879
Allele	G	127/283 (52.5/53.8)	1.00		42/283 (51.2/53.8)	1.00		85/283 (53.1/53.8)	1.00	
	C	115/243 (47.5/46.2)	1.06 (0.78–1.43)	0.733	40/243 (48.8/46.2)	1.11 (0.70–1.77)	0.663	75/243 (46.9/46.2)	1.03 (0.72–1.47)	0.880

*Adjusted for age and gender status.

& *P*<0.05.

*P* for Chi-square analysis or Fisher's exact test.

We further classified the study subjects into T-ALL and B-ALL (Table 3). The rs10519612 C allele carrier frequency was higher in the T-ALL patients than in controls (*P* = 0.030, OR 1.67, 95%CI 1.05–2.67). The rs10519612 CC genotype and the rs17007695 TC genotype frequencies were higher in patients with T-ALL than in controls (*P* = 0.039, OR 2.68, 95%CI 1.05–6.83; *P* = 0.049, OR 2.28, 95%CI 1.01–5.17). The distribution of rs10519612 and rs17007695 genotypes were significantly different between B-ALL patients and controls (*P* = 0.049; *P* = 0.003), while only the distribution of rs10519612, but not rs17007695 genotypes were different between T-ALL patients and controls (*P* = 0.041; *P* = 0.095). An increased risk for B-ALL was found for the rs17007695 TC genotype (*P* = 0.004, OR 2.40, 95%CI 1.32–4.36).

We also studied whether the *IL-15* SNPs were different in BCR-ABL B-ALL patients and other B-ALL patients (Table S1). Only the distribution of rs17007695 genotypes in BCR-ABL B-ALL patients (*P* = 0.004) and rs10519612 genotypes in other B-ALL patients (*P* = 0.041) was significantly different from the controls. Increased risks were found for the rs17007695 TC genotype both in BCR-ABL patients (OR 4.35, 95%CI 1.22–15.55) and other B-ALL patients (OR 1.99, 95%CI 1.03–3.85).

### Haplotypes ACAC, CAGT and CCAT were significantly associated with adult ALL (especially B-ALL), while haplotype CCAT conferred susceptibility to T-ALL

**Figure 1:**
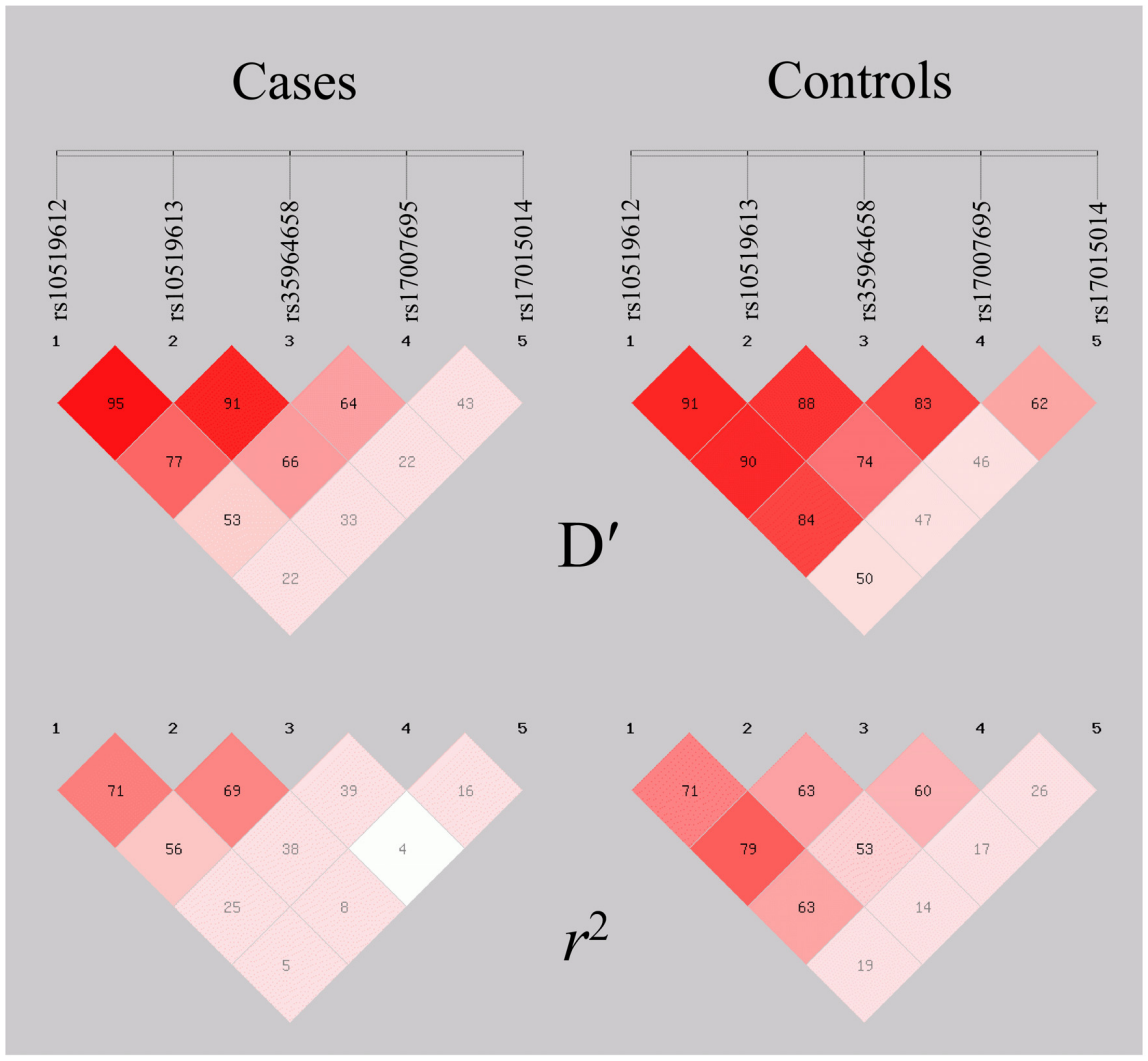
LD maps of the five SNPs of *IL-15*. LD maps of the five SNPs of *IL-15* for ALL cases and controls were generated by SHEsis program. For each SNP, D' and pair r^2^ values were shown in diamonds in two groups, including ALL patients and healthy controls.

D' and r^2^ for these five polymorphisms were calculated according to the genotyping data reported in Table 3 and Table 4. Their LD maps measured by D' and r^2^ in cases and controls were shown in Figure 1. 5-SNP haplotypes were reconstructed according to the genotyping data in cases and controls. The frequencies of haplotypes ACACC, CAGCG and CCATG were higher in ALL patients (OR 2.93, 95%CI 1.26–6.82, *P* = 0.010; OR 2.17, 95%CI 1.15–4.11, *P* = 0.016; OR 5.27, 95%CI 1.71–16.26, *P* = 0.001). When the patients were further classified into T-ALL and B-ALL, the frequencies of haplotypes ACACC and CAGCG were also higher in B-ALL patients (OR 2.89, 95%CI 1.12–7.46, *P* = 0.023; OR 2.09, 95%CI 1.00–4.34, *P* = 0.046) and the frequency of haplotype CCATG was significantly higher in T-ALL patients compared with controls (OR 8.67, 95%CI 2.34–32.11, *P*<0.001) (Table S2).

**Table 4:** Haplotype frequencies of *IL-15* in ALL cases and controls*.

Haplotype†	All Subjects			T-ALL Subjects			B-ALL Subjects		
	Frequencies§	OR(95%CI)	*P*	Frequencies§	OR(95%CI)	*P*	Frequencies§	OR(95%CI)	*P*
A C A T	40.2/53.2	1.00		39.3/53.2	1.00		40.9/53.2	1.00	
A C A C	6.7/2.7	3.33 (1.57–7.08)	0.001	5.4/2.7	2.50 (0.78–8.05)	0.113	7.5/2.7	3.75 (1.66–8.49)	0.001
C A G C	31.2/29.2	1.40 (0.98–2.01)	0.067	34.4/29.2	1.59 (0.92–2.74)	0.092	29.9/29.2	1.34 (0.87–2.04)	0.181
C A G T	7.2/3.7	2.61 (1.30–5.23)	0.005	4.4/3.7	1.84 (0.59–5.75)	0.286	8.4/3.7	2.99 (1.41–6.38)	0.003
C C A T	4.9/1.0	7.00 (2.40–20.38)	<0.001	6.2/1.0	8.75 (2.40–31.86)	<0.001	4.1/1.0	5.25 (1.55–17.74)	0.003

*Haplotypes with frequencies of more than 5% were included.

† Four SNPs alleles from left to right (rs10519612, rs10519613, rs35964658 and rs17007695) were used for reconstruction of haplotypes.

§ Haplotype frequencies (%) in patients and healthy controls.

Four polymorphisms consisting of rs10519612, rs10519613, rs35964658 and rs17007695 were in a LD block. Accordingly, 4-SNP haplotypes (rs10519612, rs10519613, rs35964658 and rs17007695) were reconstructed according to the genotyping data in ALL patients and controls. Haplotypes with frequencies ≥5% were shown in Table 4. The four common haplotype alleles represented 89.8% of the chromosomes in the controls. The most frequent haplotypes observed in patients and controls were haplotype ACAT (40.2%, 53.2%) and CAGC (31.2%, 29.2%). The frequencies of haplotypes ACAC, CAGT and CCAT were significantly higher in ALL patients compared with healthy controls (OR 3.33, 95%CI 1.57–7.08, *P* = 0.001; OR 2.61, 95%CI 1.30–5.23, *P* = 0.005; OR 7.00, 95%CI 2.40–20.38, *P*<0.001). When the data were analyzed separately, the frequencies of haplotypes ACAC, CAGT and CCAT were only significantly higher in B-ALL patients compared with healthy controls (OR 3.75, 95%CI 1.66–8.49, *P* = 0.001; OR 2.99, 95%CI 1.41–6.38, *P* = 0.003; OR 5.25, 95%CI 1.55–17.74, *P* = 0.003). However, carriage of the CCAT haplotype conferred a 9-fold higher susceptibility to T-ALL (OR 8.75, 95%CI 2.40–31.86, P<0.001).

## Discussion

IL-15 is a proinflammatory cytokine that promotes the proliferation of T, B and NK cells and induction of cytolytic effector cells [27], [35], [36]. It plays an important role not only in immune system but also in the growth and survival of immature hematopoietic cells [21]–[23], which could be the reason for its role in pathogenesis of leukemic cells [24], [25]. Some studies have already demonstrated that IL-15 can protect hematologic tumors from drug induced apoptosis *in vitro* [25], and high expression of IL-15 correlates with CNS disease in childhood ALL [26]. Several *IL-15* SNPs which were associated with minimal residual disease (MRD) have been linked to enhanced IL-15 transcription/translation efficiency *in vitro* [37]. Five SNPs located in the *IL-15* locus identified by genome-wide scan significantly associated with childhood ALL treatment response. Therefore, IL-15 plays a role in treatment response in childhood ALL [31]. In adult ALL, the expression of IL-15 was correlated with the immunophenotypes of ALL, patients with T-ALL and B-cell precursor (BCP)-ALL have different expression levels of IL-15 [38]. In the current study, we identified two SNPs (rs10519612, rs17007695) that were associated with the risk for adult ALL. In the haplotype analysis, we further concluded that haplotypes ACAC, CAGT and CCAT were significantly associated with adult ALL (especially B-ALL), while haplotype CCAT conferred susceptibility to T-ALL.

When we evaluated each of the selected SNPs' genotypes separately, the rs10519612 CC genotype and rs17007695 TC genotype were found to be associated with the increased risk; the other SNPs had no significant correlation with the risk for adult ALL. We demonstrated that the presence of the rs10519612 CC genotype and the rs17007695 TC genotype increased the risk of ALL about two folds. When T-ALL and B-ALL were analyzed separately, rs10519612 CC genotype and the rs17007695 TC genotype conferred risk for T-ALL (2.68 fold and 2.28 fold increase), while only the rs17007695 TC genotype was associated with B-ALL (2.4 fold increase). In B-ALL study, the distribution of rs10519612 CC and rs17007695 TC genotypes were different compared with controls, while only the distribution of rs10519612 CC genotypes were different in T-ALL.

Although five SNPs have been reported to be associated with childhood ALL treatment response [31], we only found two of them correlated with adult ALL in the present study. Out of total 121 ALL patients recruited in this study, 48 were treated relatively uniformly with induction regimen. Among those, 34 achieved CR (T-ALL 5, B-ALL 29), 4 achieved PR (T-ALL 3, B-ALL 1), and ten of them were not responding (NR, T-ALL 8, B-ALL 2). We performed association studies of the SNPs of *IL-15* gene with the treatment responses, and none of them was significantly associated with the treatment responses. We have not analyzed the association of these SNPs with the disease prognosis and treatment responses, which would be extremely meaningful in future studies. The combined analysis of SNPs in *IL-15* and other genes may also be needed since the polymorphisms in *IL-15* might not serve as a sole risk factor for the disease prognosis. Moreover, our study may have certain limitations because of the study design. Selection bias and/or systematic error may occur because the cases were from hospital and the controls were from community. Some factors which may interact with genotype or act as potential confounders in analysis such as information of MRD is not available in our case-control studies.

In the present study, we demonstrated that the polymorphisms of *IL-15* genes were associated with adult ALL. Although whether the SNPs that were significantly associated with the disease are functional needs to be further investigated, our results presented here clearly suggested that IL-15 play a role for in the tumorigenesis of adult ALL and may lead to the perspective for IL-15 as a novel therapeutic target in adult ALL.

## Supporting Information

Table S1 Genotype and allele frequencies of IL-15 SNPs among ALL cases and controls and associations with risk of ALL.
(0.09 MB DOC)

Table S2 Haplotype frequencies of IL-15 in ALL cases and controls.
(0.04 MB DOC)
