# Supplementary material for: The Role of Interleukin-15 Polymorphisms in Adult Acute Lymphoblastic Leukemia
Source: PLoS One. 2010 Oct 25;5(10):e13626. doi: 10.1371/journal.pone.0013626 (PMC2963612; doi:10.1371/journal.pone.0013626)
Supplement: Table S1 — Genotype and allele frequencies of IL-15 SNPs among ALL cases and controls and associations with risk of ALL. (0.09 MB DOC) [file pone.0013626.s001.doc]

Table S1 Genotype and allele frequencies of *IL-15* SNPs among ALL cases and controls and associations with risk of ALL

| SNP |  | BCR-ABL B-ALL Subjects (Cases / controls) | | |  | Other B-ALL Subjects (Cases / controls) | | |
| --- | --- | --- | --- | --- | --- | --- | --- | --- |
|  |  | n (%) | OR (95% CI)^*^ | *P* |  | n (%) | OR (95% CI)^*^ | *P* |
| rs10519612 |  |  |  |  |  |  |  |  |
| Genotype | AA | 7 / 91 (35.0 / 34.6 ) | 1.00 |  |  | 21 / 91 (35.0 / 34.6) | 1.00 |  |
|  | AC | 9 / 135 (45.0 / 51.3) | 0.81 (0.29-2.27) |  |  | 23 / 135 (38.3 / 51.3) | 0.70 (0.36-1.35) |  |
|  | CC | 4 / 37 (20.0 / 14.1) | 1.23 (0.33-4.53) | 0.741 |  | 16 / 37 (26.7 / 14.1) | 1.72 (0.80-3.71) | 0.041 |
| Allele | A | 23 / 317 (57.5 / 60.3) | 1.00 |  |  | 65 / 317 (54.2 / 60.3) | 1.00 |  |
|  | C | 17 / 209 (42.5 / 39.7) | 1.12 (0.59-2.15) | 0.731 |  | 55 / 209 (45.8 / 39.7) | 1.28 (0.86-1.91) | 0.220 |
| rs10519613 |  |  |  |  |  |  |  |  |
| Genotype | CC | 6 / 103 (30.0 / 39.2) | 1.00 |  |  | 22 / 103 (36.7 / 39.2) | 1.00 |  |
|  | CA | 13 / 131 (65.0 / 49.8) | 1.55 (0.56-4.27) |  |  | 26 / 131 (43.3 / 49.8) | 0.89 (0.47-1.67) |  |
|  | AA | 1 / 29 (5.0 / 11.0) | 0.47 (0.05-4.18) | 0.390 |  | 12 / 29 (20.0 / 11.0) | 1.80 (0.78-4.12) | 0.165 |
| Allele | C | 25 / 337 (62.5 / 64.1) | 1.00 |  |  | 70 / 337 (58.3 / 64.1) | 1.00 |  |
|  | A | 15 / 189 (37.5 / 35.9) | 1.07 (0.55-2.08) | 0.842 |  | 50 / 189 (41.7 / 35.9) | 1.27 (0.85-1.91) | 0.240 |
| rs35964658 |  |  |  |  |  |  |  |  |
| Genotype | AA | 4 / 85 (21.1 / 32.3) | 1.00 |  |  | 17 / 85 (28.8 / 32.3) | 1.00 |  |
|  | AG | 12 / 142 (63.2 / 54.0) | 1.64 (0.51-5.32) |  |  | 29 / 142 (49.2 / 54.0) | 0.97 (0.50-1.89) |  |
|  | GG | 3 / 36 (15.8 / 13.7) | 1.62 (0.34-7.72) | 0.594 |  | 13 / 36 (22.0 / 13.7) | 1.70 (0.74-3.90) | 0.272 |
| Allele | A | 20 / 312 (52.6 / 59.3) | 1.00 |  |  | 63 / 312 (53.4 / 59.3) | 1.00 |  |
|  | G | 18 / 214 (47.4 / 40.7) | 1.31 (0.68-2.54) | 0.419 |  | 55 / 214 (46.6 / 40.7) | 1.27 (0.85-1.90) | 0.238 |
| rs17007695 |  |  |  |  |  |  |  |  |
| Genotype | TT | 3 / 104 (15.0 / 39.5) | 1.00 |  |  | 15 / 104 (25.0 / 39.5) | 1.00 |  |
|  | TC | 17 / 124 (85.0 / 47.1) | 4.35 (1.22-15.55) ^＆^ |  |  | 38 / 124 (63.3 / 47.1) | 1.99 (1.03-3.85) ^＆^ |  |
|  | CC | 0 / 35 (0.0 / 13.3) | 0.00 | 0.004 |  | 7 / 35 (11.7 / 13.3) | 1.25 (0.46-3.38) | 0.065 |
| Allele | T | 23 / 332 (57.5 / 63.1) | 1.00 |  |  | 68 / 332 (56.7 / 63.1) | 1.00 |  |
|  | C | 17 / 194 (42.5 / 36.9) | 1.27 (0.66-2.43) | 0.479 |  | 52 / 194 (43.3 / 36.9) | 1.31 (0.88-1.96) | 0.189 |
| rs17015014 |  |  |  |  |  |  |  |  |
| Genotype | GG | 4 / 75 (20.0 / 28.5) | 1.00 |  |  | 17 / 75 (28.3 / 28.5) | 1.00 |  |
|  | GC | 11 / 133 (55.0 / 50.6) | 1.45 (0.44-4.75) |  |  | 32 / 133 (53.3 / 50.6) | 1.03 (0.53-1.98) |  |
|  | CC | 5 / 55 (25.0 / 20.9) | 1.69 (0.43-6.63) | 0.704 |  | 11 / 55 (18.3 / 20.9) | 0.88 (0.38-2.04) | 0.890 |
| Allele | G | 19 / 283 (47.5 / 53.8) | 1.00 |  |  | 66 / 283 (55.0 / 53.8) | 1.00 |  |
|  | C | 21 / 243 (52.5 / 46.2) | 1.29 (0.68-2.45) | 0.441 |  | 54 / 243 (45.0 / 46.2) | 0.95 (0.64-1.42) | 0.812 |

^*^Adjusted for age and gender status. ^＆^*P*＜0.05

*P* for Chi-square analysis or Fisher's exact test.
