# Supplementary material for: The Role of Interleukin-15 Polymorphisms in Adult Acute Lymphoblastic Leukemia
Source: PLoS One. 2010 Oct 25;5(10):e13626. doi: 10.1371/journal.pone.0013626 (PMC2963612; doi:10.1371/journal.pone.0013626)
Supplement: Table S2 — Haplotype frequencies of IL-15 in ALL cases and controls. (0.04 MB DOC) [file pone.0013626.s002.doc]

Table S2 Haplotype frequencies of *IL-15* in ALL cases and controls^*^

| Haplotype^†^ | All Subjects | | |  | T-ALL Subjects | | |  | B-ALL Subjects | | |
| --- | --- | --- | --- | --- | --- | --- | --- | --- | --- | --- | --- |
|  | Frequencies^§^ | OR(95%CI) | *P* |  | Frequencies^§^ | OR(95%CI) | *P* |  | Frequencies^§^ | OR(95%CI) | *P* |
| A C A T G | 29.9 / 39.6 | 1.00 |  |  | 28.7 / 39.6 | 1.00 |  |  | 30.7 / 39.6 | 1.00 |  |
| A C A C C | 5.1 / 2.3 | 2.93 (1.26-6.82) | 0.010 |  | 5.2 / 2.3 | 2.89 (0.86-9.67) | 0.073 |  | 5.1 / 2.3 | 2.89 (1.12-7.46) | 0.023 |
| A C A T C | 10.5 / 13.7 | 1.02 (0.60-1.73) | 0.950 |  | 10.9 / 13.7 | 1.08 (0.48-2.44) | 0.847 |  | 10.4 / 13.7 | 0.96 (0.52-1.80) | 0.906 |
| C A G C C | 23.2 / 24.1 | 1.27 (0.84-1.92) | 0.261 |  | 26.1 / 24.1 | 1.43 (0.77-2.68) | 0.258 |  | 22.2 / 24.1 | 1.19 (0.73-1.95) | 0.476 |
| C A G C G | 8.3 / 5.1 | 2.17 (1.15-4.11) | 0.016 |  | 8.1 / 5.1 | 2.25 (0.88-5.71) | 0.082 |  | 8.3 / 5.1 | 2.09 (1.00-4.34) | 0.046 |
| C C A T G | 3.7 / 1.0 | 5.27 (1.71-16.26) | 0.001 |  | 6.1 / 1.0 | 8.67 (2.34-32.11) | <0.001 |  | 2.3 / 1.0 | 3.47 (0.90-13.40) | 0.056 |

^*^Haplotypes with frequencies of more than 5% were included.

^†^Five SNPs alleles from left to right (rs10519612, rs10519613, rs35964658, rs17007695 and rs17015014) were used for reconstruction of haplotypes.

^§^Haplotype frequencies (%) in patients and healthy controls.
